# Anopheline antiplatelet protein from mosquito saliva regulates blood feeding behavior

**DOI:** 10.1038/s41598-019-39960-2

**Published:** 2019-02-28

**Authors:** Ashekul Islam, Talha Bin Emran, Daisuke S. Yamamoto, Mitsuhiro Iyori, Fitri Amelia, Yenni Yusuf, Ririka Yamaguchi, Md. Shah Alam, Henrique Silveira, Shigeto Yoshida

**Affiliations:** 1 Laboratory of Vaccinology and Applied Immunology, Kanazawa University School of Pharmacy, Kakuma-machi, Kanazawa, 920-1192 Japan; 2 Division of Medical Zoology, Department of Infection and Immunity, Jichi Medical University, 3311-1 Yakushiji, Shimotsuke, 329-0431 Japan; 3 Laboratory of Ecology, Graduate School of Natural Science and Technology, Kanazawa University, Kanazawa, 920-1192 Japan; 4 Laboratory of Vector-borne diseases and Pathogens, Global Health and Tropical Medicine, Instituto de Higiene e Medicina Tropical, Universidade Nova de Lisboa, Lisbon, 1099-085 Portugal

## Abstract

The saliva of hematophagous arthropods is enriched with a complex mixture of antihemostatic molecules, the biological functions of which are largely unknown. Anopheline antiplatelet protein (AAPP) from malaria vector mosquito exhibits strong antiplatelet activity when bound directly to host collagen by its C-terminus and through its N-terminus with Ca^2+^-binding activity. To investigate the biological functions of AAPP in blood feeding behavior and malaria transmission, we generated transgenic *Anopheles stephensi* mosquito lines expressing anti-AAPP antibody single-chain fragment (scFv) in their salivary glands. The AAPP-specific collagen-binding activity was completely abolished by AAPP-scFv complex formation in the saliva. Probing and prediuresis time, feeding success, blood meal size, and fecundity, which are all fitness characteristics, were significantly reduced in the transgenic mosquitoes. However, oocysts number in these mosquitoes were not significantly reduced following blood meal intake from *Plasmodium berghei*-infected mice. These results show that although AAPP plays an important role in mosquito blood feeding, its neutralizing activity did not affect sporogonic development in our laboratory model, but its high fitness cost would pose a survival risk for parasite-infected mosquitoes in nature.

## Introduction

Blood-sucking insects utilize a diverse array of feeding strategies on their hosts, which enables them to counteract the obstacles associated with host homeostasis^1^. Mosquitoes search for a suitable blood vessel moving their mouthparts into the skin during the probing phase. Successful probing along with the injection of saliva components known to enable the mosquito to obtain a blood meal and counteract host homeostasis^2^. Several studies have reported on the potent and pleiotropic effects of saliva proteins from hematophagous arthropods, which include anti-coagulation, vasodilation and anti-inflammation^3^. The roles played by these proteins in blood feeding have been demonstrated through the use of *in vitro* biochemical analyses even though the biological relevance of these saliva proteins on mosquito function, such as blood feeding propensity, fecundity, egg hatching rates, and viability remains largely unknown. Furthermore, saliva components delivered to the bite site with vector-borne pathogen have been shown to modulate vertebrate immune responses^4^. For example, tick derived saliva factors appear to inhibit inflammatory cytokine secretion^5^; SAAG-4 from *Aedes aegypti* mosquito saliva has the potential to alter the Th-profile of the bite-induced immune responses^6^; and sand fly saliva has a caspase-dependent, pro-apoptotic effect on neutrophils^7^. The main effect of immunomodulatory saliva components in regard to infection is appear to be temporary and local that allow the vector to feed, resulting in establishing an infection^8^.

In mosquitoes, transgenesis-based gene silencing and saliva protein inactivation are potentially useful methods for analyzing the effects of saliva proteins on mosquito physiology. We have established a female salivary gland-specific transgene expression system in transgenic *Anopheles stephensi* mosquitoes using the promoter region of the anopheline antiplatelet protein (AAPP) gene, which encodes an inhibitor of collagen-induced platelet aggregation in the salivary glands^9–11^. Using this system, several foreign effector genes encoding the SP15 saliva protein of the *Phlebotomus papatasi* sand fly^12^, the repeat region of the *Plasmodium berghei* circumsporozoite protein (CSP)^13,14^ and the anti-*P. falciparum* circumsporozoite protein (PfCSP) single-chain Ab (scFv)^15^ have been functionally expressed in the salivary glands as a component of saliva. This system could also be used for gene silencing or protein inactivation for studying the effects of saliva proteins on the ecological attributes of mosquitoes. Recently, Chagas *et al*. (2014) used mosquito transgenesis for gene silencing with RNA interference (RNAi) in *Aedes aegypti*. The authors showed that silencing the *Aegyptin* gene, which encodes a homolog of AAPP, significantly reduced its cognate mRNA and protein levels in the salivary glands of female mosquitoes, resulting in prolonged probing time and reduced feeding quality and quantity^16^.

In the present study, instead of transgenic RNAi, we used a transgenesis-based protein inactivation approach to explore the functions of AAPP, which is the predominant saliva protein in the main Asian malaria vector mosquito *An. stephensi*^17^. AAPP and Aegyptin belong to a member of 30-kDa GE-rich salivary gland protein family with comparable modes of collagen-binding action^10,18,19^. We have previously reported^20^ that AAPP possesses four cysteine residues at its C-terminus and exhibits strong antiplatelet activity. AAPP is also an acidic secretory protein with an isoelectric point (pI) of 3.8, and a GE-rich region containing 10 unique repeats of a 6-amino acid unit (GEEGGA) and related sequences at its N-terminus. Because several Ca^2+^-binding proteins are known to have clusters of acidic amino acids^21^, AAPP might possess Ca^2+^-binding properties. Therefore, we generated transgenic (TG) *Anopheles stephensi* mosquito lines that express anti-AAPP scFv in their salivary glands. Functional inactivation of AAPP via expression of an anti-AAPP scFv in the salivary glands was found to completely abolish the collagen-binding activity of AAPP. Consequently, a significant increase in probing and prediuresis time, reduction in feeding success, blood meal size, and fecundity were observed in the TG mosquitoes, as compared with their wild-type (WT) counterparts. Sporogonic development in the TG mosquitoes showed no significant reduction in terms of oocysts number following blood meals on the *P. berghei*-infected mice. These results indicate that AAPP plays an important role in facilitating blood feeding, but impairment of blood feeding behavior did not affect the malaria vectorial capacity (sporogonic development) in our laboratory model.

## Results

### AAPP is a Ca^2+^-binding protein

**Figure 1:**
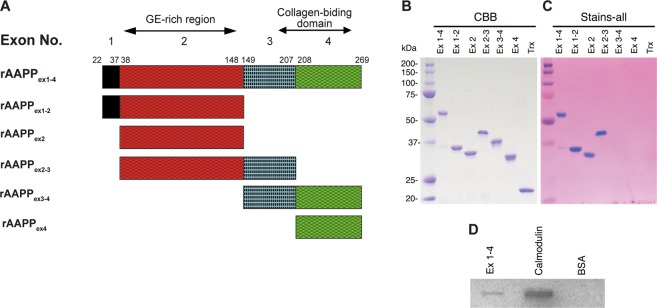
Expression and purification of AAPP truncated forms and Ca^2+^ binding assay. (**A**) Schematic representation of AAPP truncated forms. The *AAPP* gene is encoded by four exons. A series of truncated rAAPPs according to the exon arrangement were produced in *E. coli* expression system. The GE-rich region and collagen binding domain are shown. (**B**) SDS-PAGE analyses of AAPP truncated forms. (**C**) Comparative staining of acidic proteins with Stains-All of AAPP truncated forms. (**D**) Calcium binding assay of AAPP truncated form on nitrocellulose membrane after SDS electrophoresis with the reference of calmodulin. Protein bands are shown after autoradiography.

AAPP, a predominant saliva protein in the malaria vector *An. stephensi*^10^, exhibits strong antiplatelet activity by binding directly to collagen and subsequently blocking platelet aggregation via its C-terminus conformational domain, which contains four cysteine residues^20^. AAPP also has another characteristic feature of acidic secretory proteins (pI = 3.8) in that, as mentioned above, it contains a GE-rich region containing 10 unique repeats of a 6-amino acid unit (GEEGGA) or related sequences at the N-terminus. Therefore, as several Ca^2+^-binding proteins are known to have clusters of acidic amino acid residues, we examined whether AAPP has Ca^2+^ binding properties. Because the AAPP gene is encoded by four exons, the *Escherichia coli* expression system was used as reported previously to produce a series of truncated AAPP recombinant proteins (rAAPPs) according to the exon arrangement^20^ depicted in Fig. 1A. Purified rAAPPs were stained with Stains-All reagent, which has previously been used to identify Ca^2+^-binding activity^22^. Truncated rAAPP_ex1–4_, rAAPP_ex1–2_, rAAPP_ex2_ and rAAPP_ex2–3_ were clearly stained blue with Stains-All, unlike rAAPP_ex3–4_ and rAAPP_ex4_ (Fig. 1B,C). This result indicates that the highly negatively charged GE-rich region encoded by exon 2 contains Ca^2+^ binding sites (Fig. 1A). The ^45^Ca^2+^ overlay assay directly evidences the Ca^2+^ binding property of rAAPP_ex1–4_, which is estimated to be eight times lower than that of calmodulin when equimolar amounts were tested (Fig. 1D). No Ca^2+^-binding property was observed for the bovine serum albumin (BSA) control.

### Establishing transgenic mosquitoes

**Figure 2:**
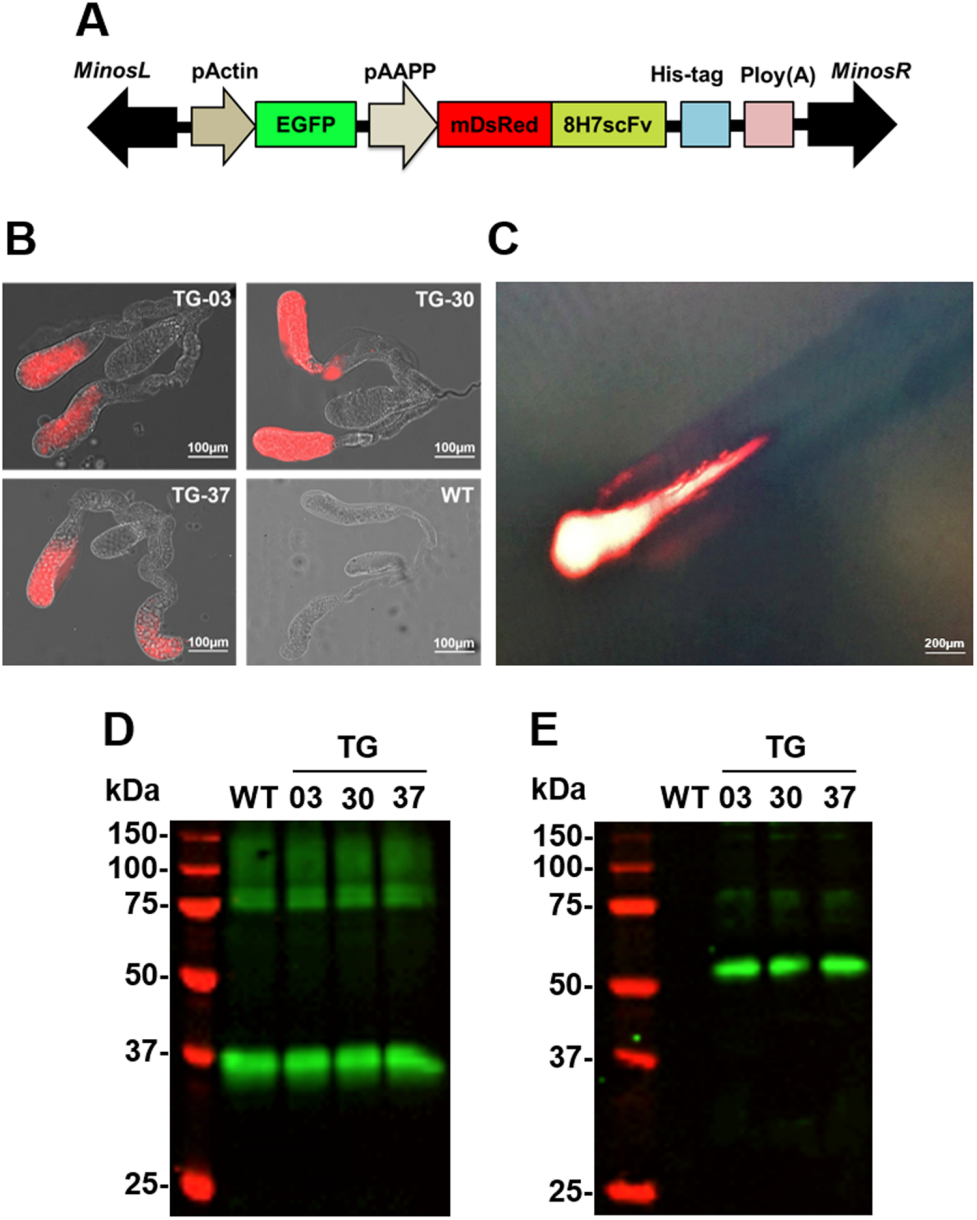
Establishment of the mDsRed-8H7scFv TGs. (**A**) Schematic representation of *Minos*-based transformation vector harboring 8H7scFv gene fused with monomeric DsRed (mDsRed-8H7scFv) expressing under the control of aapp promoter (*pAAPP*). The transformation marker, EGFP is expressed under the control of midgut driven actin5c promoter (*pActin*). (**B**) Overlay images of the mDsRed fluorescence of the salivary glands. Scale bars: 100 µm. (**C**) Observation of salivation under fluorescence microscope. Scale bars: 200 µm. (**D,E**) Detection of mDsRed-8H7scFv and AAPP by immunoblotting. Lysates of dissected salivary glands from WT and TG mosquitoes were probed with anti-AAPP mAb, 28B8 (**D**) or anti-mDsRed polyclonal Ab (**E**).

We have previously tested several recombinant truncated genes carrying different combinations of the four exons to locate the precise collagen binding sites in AAPP. Of them, exons 3 and 4 are absolutely required for collagen-AAPP interactions^20^. We also obtained a series of anti-AAPP monoclonal Abs (mAbs)^23^ from which the 8H7 mAb was found to be a potent inhibitory Ab capable of blocking the binding of AAPP to collagen, while the 28B8 mAb (Supplementary Fig. S1) was not. The crystal structure of the collagen-binding domain of AAPP has been solved with bound 8H7 Fab^23^. The heavy chain (V_H_) and light chain (V_L_) gene fragments encoding the 8H7 mAb were cloned from 8H7 hybridoma cells by reverse-transcription-PCR as described previously^23^. The nucleotide sequence data for the V_H_ and V_L_ genes have been deposited in GenBank^®^ database (https://www.ncbi.nlm.nih.gov/genbank/) under the accession numbers AB903029 and AB903030. A *Minos-*based transformation vector harboring a gene cassette encoding 8H7scFv fused to mDsRed through Gly_4_Ser × 3 linkers with a hexahistidine-tag at the C-terminus under the control of the female salivary gland-specific *aapp* promoter, was constructed (Fig. 2A) and injected together with a *Minos* helper plasmid^14^ into *An. stephensi* embryos. Three independent TG mosquito lines (TG-03, TG-30, and TG-37) were established and stably maintained by intercrossing with the transgenic siblings. Fluorescence microscopy revealed strong red fluorescence in the distal-lateral lobes of the dissected female salivary glands from the TG mosquitoes (Fig. 2B). We also observed using fluorescence microscopy that the mDsRed-8H7scFv protein was released from the proboscis as a saliva component (Fig. 2C). This expression pattern is consistent with our previous transgenic lines established using the same *aapp* promoter^12,14,15^.

### Gene dose determination in transgenic mosquitoes

The copy numbers of the transgenes to be inserted into the genomes of three TG mosquito lines was determined by quantitative real-time PCR (qPCR). The salivary gland-specific AAPP promoter gene *(pAAPP)* was selected as an internal reference single-copy gene because it is highly conserved. The gene dose ratio (GDR) values obtained are summarized in the Supplementary Figure (Supplementary Fig. S2). As can be observed, the *pAAPP* gene was the same (i.e., remained a single copy) in the WT mosquitoes, while duplicate gene copies were clearly separated in the TG mosquito lines. These results indicate that there was single copy insertion per genome in all the TG mosquito lines.

### Complex formation between 8H7scFv and AAPP in the salivary glands

**Figure 3:**
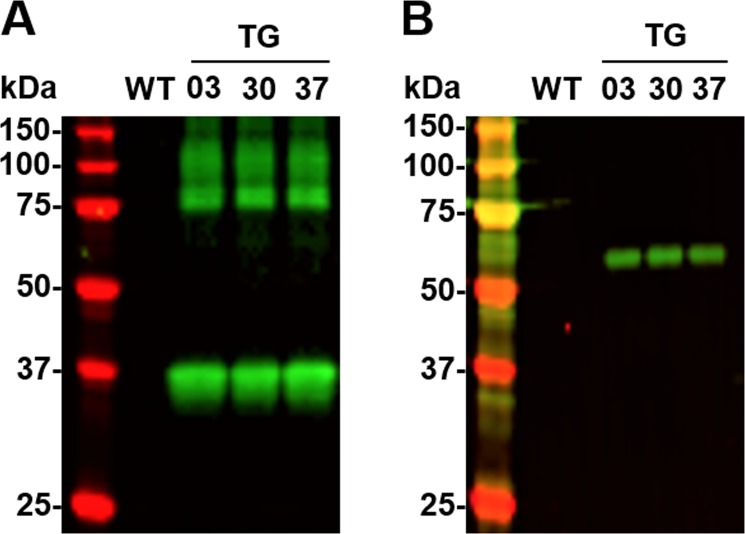
Ni-NTA pull-down assay. (**A,B**) Demonstration of mDsRed-8H7scFv and AAPP by immunoblotting. Salivary proteins eluted from the Ni-NTA resin were probed with anti-AAPP mAb, 28B8 (**A**) or anti-mDsRed polyclonal Ab (**B**).

Immunoblots probed with the 28B8 mAb revealed that AAPP (M*r* = 37 kDa) was detectable in the salivary gland lysates from both WT and TG mosquitoes at similar amounts (Fig. 2D, lanes WT, and TG). A 54.6 kDa protein corresponding mDsRed-8H7scFv was detected in lysates from the dissected salivary glands from the TG mosquitoes using the anti-mDsRed polyclonal Ab (Fig. 2E, lane; TG). We next performed a pull-down assay to detect mDsRed-8H7scFv─AAPP in its complexed form. Because the mDsRed-8H7scFv protein has a hexahistidine-tag at its C-terminus, Ni-NTA resin was used to perform the pull-down assays with the salivary gland lysates. After elution from the Ni-NTA resin, AAPP (Fig. 3A, TG lanes) and mDsRed-8H7scFv (Fig. 3B, TG lanes) were detected in TG mosquitoes by the anti-AAPP mAb (28B8) and the anti-DsRed polyclonal Ab, respectively, whereas no signals were detected in the WT mosquitoes (Fig. 3A,B; WT lanes). These results clearly revealed specific binding between the two proteins and resulted in forming a complex.

### Complexation of mDsRed-8H7scFv and AAPP severely impairs AAPP–collagen binding

To investigate whether the mDsRed-8H7scFv**–**AAPP complex could block the collagen-binding activity of AAPP and abrogate its function(s), we collected saliva from the proboscises of 20 female mosquitoes during salivation (Fig. 2C). Salivary gland homogenates were also prepared from dissected mosquitoes. Red fluorescence was observed in both the saliva and salivary gland homogenates (Supplementary Fig. S3). The collagen-binding enzyme-linked immunosorbent assay (ELISA) showed that the saliva from 20 mosquitoes from each TG line completely abrogated the AAPP-collagen binding activity (detection limit <0.069 ng), whereas the saliva from 20 WT mosquitoes retained AAPP-mediated collagen-binding activity (28.4 ± 0.57 ng) (Table 1). The salivary gland homogenates from each TG line retained a trace of collagen-binding activity (9.95–11.37 ng), which is 23-fold lower than that for WT mosquitoes (232.7 ± 1.87 ng), indicating that minute amounts of unbound AAPP existed in the TG lines.

**Table 1:** Functional depletion of AAPP by forming complex with mDsRed-8H7scFv in the saliva and salivary glands^a^.

Group	WT	TG-03	TG-30	TG-37
Sample				
Saliva (ng)	28.4 ± 0.57	N.D.	N.D.	N.D.
Salivary glands (ng)	232.7 ± 1.87	11.37 ± 0.95	9.95 ± 0.38	10.5 ± 0.30

^a^A live female mosquito was held by forceps and its proboscis was placed into PBS (pH 7.4) for 3 min to collect saliva. Alternatively, the salivary glands were dissected and homogenized. Saliva and salivary gland lysates were obtained from a total of 20 mosquitoes per group, and their red fluorescence was observed (Supplementary Fig. S3). These samples were applied for collagen-binding ELISA to measure the amount of AAPP capable of binding to collagen. Trx-AAPP_ex1–4_ was used for quantification of AAPP.

N.D., Not detected (detection limit < 0.069 ng).

### Functional depletion of AAPP prolongs probing and prediuresis times

**Figure 4:**
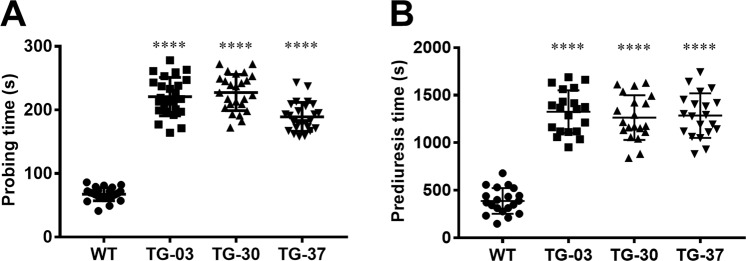
AAPP depletion prolongs probing and prediuresis time in TG mosquitoes. (**A,B**) Probing time (**A**) and prediuresis time (**B**) were determined in both WT and TG mosquitoes. Each dot represent a single female mosquito (n = 25). Mosquitoes with a probing time greater than 420 seconds were not included in statistical analysis. Results were analyzed by the Dunnett’s multiple comparisons test. Horizontal bars represent the median value. *p < 0.05, **p < 0.01, ***p < 0.001, ****p < 0.0001.

To evaluate the ecological functions of AAPP, we examined probing and prediuresis time in both TG and WT mosquitoes using a mouse model. The TG mosquitoes had significantly longer probing time (range, 158–278 s) than those of the WT mosquitoes (range, 41–86 s) (Fig. 4A). The TG mosquitoes also displayed significantly longer prediuresis time (range, 839–1742 s) than those of the WT mosquitoes (range, 147–679 s) (Fig. 4B). The prolonged probing and prediuresis time observed in the TG mosquitoes were related to 8H7scFv–AAPP complex formation in the salivary glands which probably disabled the collagen-induced platelet aggregation during blood meal. However, when TG mosquitoes were offered a naïve mouse to obtain a blood meal without time restrictions, no significant effect on the mosquito’s viability was observed (Supplementary Fig. S4).

### AAPP dysfunction impairs mosquito feeding behavior

**Figure 5:**
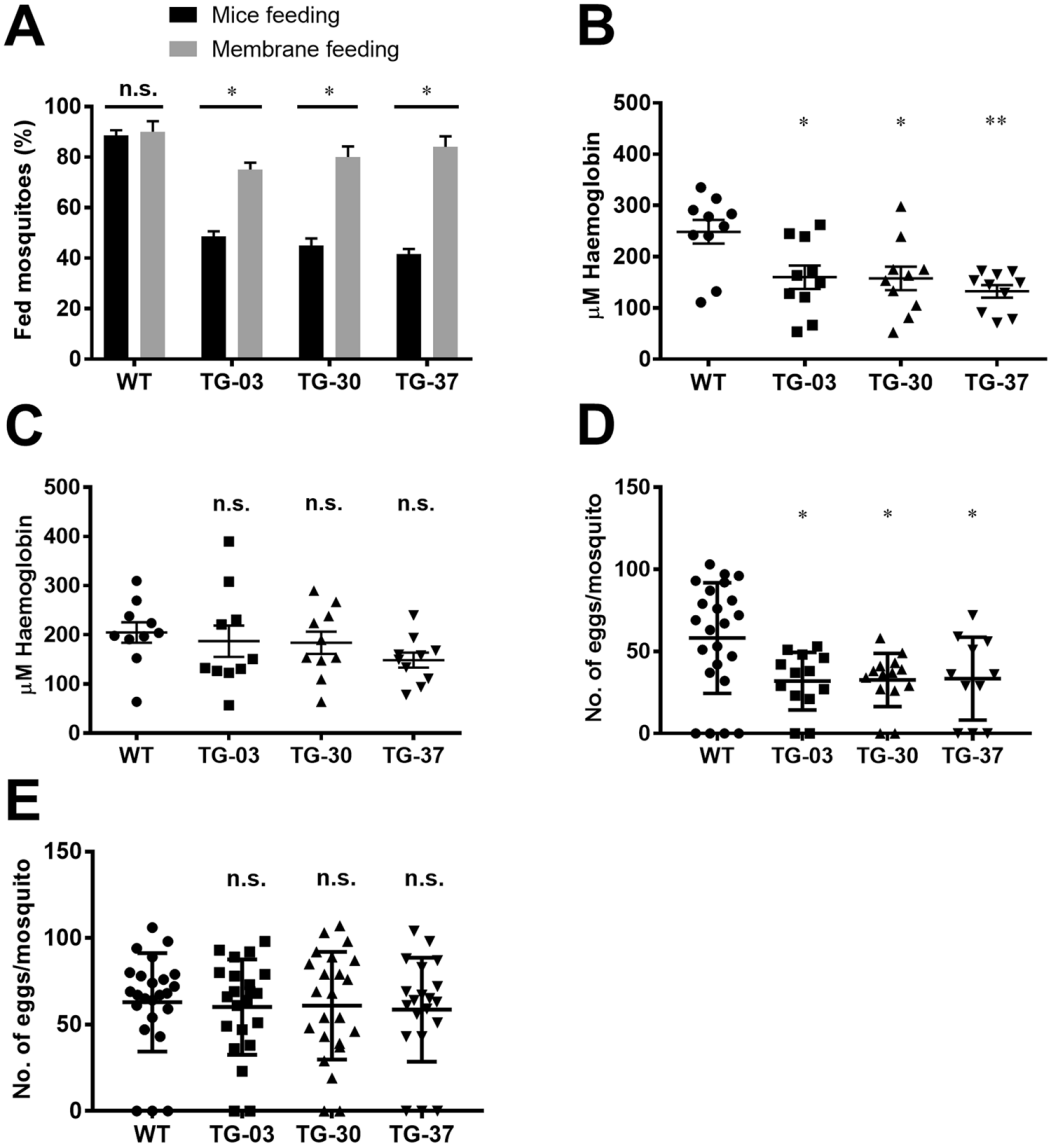
Feeding success of TG mosquitoes on direct mouse feeding and artificial membrane feeding. (**A**) Feeding success of WT and TG mosquitoes both on naïve mice (Black bar) and artificial membrane feeding (Gray bar) for 10 min blood feeding. Percentage of successful feeding were analyzed by using a *Chi*-square test with 95% CI. (**B,C**) The size of blood meal ingested by mosquitoes that fed for 10 min on mice (**B**) or artificial membrane feeder (**C**) determined by the total hemoglobin contents in the abdomens of mosquitoes. (**D,E**) Fecundity rate (egg laying) of WT and TG mosquitoes both on mice (**D**) and artificial membrane feeding (**E**) for 10 min blood feeding. Results were analyzed by the Dunnett’s multiple comparisons test. Horizontal bars represent the median value. *p < 0.05, **p < 0.01, ***p < 0.001, ****p < 0.0001. n.s., not significant.

We compared the feeding success of the TG mosquitoes in the presence (direct mouse feeding) or absence (membrane feeding assay; MFA) of collagen-induced platelet aggregation after 10 min of blood feeding. Under these conditions, the TG mosquitoes were significantly less successful in obtaining a blood meal when fed on mice (presence) compared with MFA (absence), *p* < 0.05, χ^2^ test; (Fig. 5A). We next examined the ingested blood meal size of the TG and WT mosquitoes after 10 min of blood feeding. The abdomen of each fed mosquito was homogenized and total hemoglobin was quantified with a colorimetric assay^24^. The blood volume ingested by the TG mosquitoes was significantly reduced compared with the WT mosquitoes when fed on mice (Fig. 5B). No significant difference in blood meal size was observed between the TG and WT mosquitoes when fed using MFA (Fig. 5C). Probably related to the blood meal size, fecundity was lower in the TG mosquitoes than in the WT mosquitoes when fed on mice (Fig. 5D), whereas the TG mosquitoes did not show any significant reduction in their fecundity when fed by MFA (Fig. 5E). Thus, AAPP appears to impact in blood feeding success and plays an important role in facilitating blood feeding and subsequent egg production.

### Parasite development in transgenic mosquitoes is not impaired

**Figure 6:**
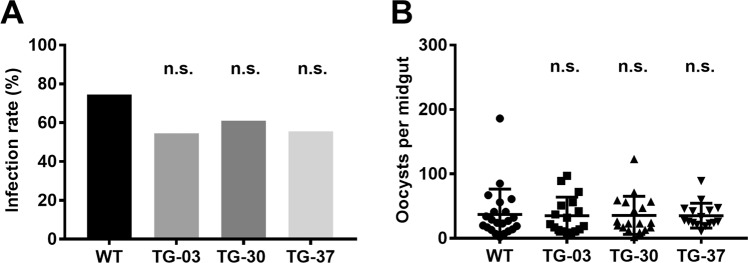
Sporogony development in TG mosquitoes. (**A,B**) WT and TG mosquitoes 5–7 days after eclosion were allowed to feed on the same *P. berghei*-infected mouse. On day 10–12, the midguts (n = 30) were dissected. Infection rate (**A**) and the number of oocysts (**B**) formed was counted. Results were analyzed by the Kruskal-Wallis test (**A**) or Dunnett’s multiple comparisons test (**B**). Horizontal bars represent the median value. n.s., not significant.

To investigate the effect of depleting AAPP activity on parasite development, TG and WT mosquitoes were allowed to feed on the same mouse that had previously been infected with *P. berghei*. There was no statistical difference in the infection rate (Fig. 6A) or oocysts number (Fig. 6B) between the TG and WT mosquitoes, indicating that AAPP has no deleterious effect on sporogonic development in the mosquito.

## Discussion

We have previously generated a malaria-refractory transgenic *Anopheles* mosquito line that expresses anti-PfCSP scFv in its salivary glands^15^. The anti-PfCSP scFv binds to transgenic sporozoites expressing PfCSP in the salivary glands, impairing their infectivity to mice. Using this scFv-mediated inhibition approach, in the present study we generated transgenic *An. stephensi* lines expressing anti-AAPP scFv in their salivary glands to investigate the role of AAPP in blood feeding, with the aim of subsequently evaluating the ecological functions of this protein in mosquitoes. The data presented here show that the collagen-binding activity of AAPP was almost completely abolished by the scFv secreted in the saliva of the TG mosquitoes. By measuring probing and prediuresis time, feeding success, blood meal size, and fecundity, we found that the TG mosquitoes showed a significant reduction in these fitness parameters when compared with the WT mosquitoes. A rapid mechanism capable of defeating host homeostasis at the probing site is a prerequisite for hematophagous arthropod survival. Prolonged probing and prediuresis time can alert vertebrate hosts to biting, resulting in feeding termination or death for blood-sucking arthropods. Therefore, faster probing or feeding time reduces the vector-host association and increases survival of blood-sucking arthropods.

AAPP, Aegyptin and their homologs, which are only conserved among mosquito species as members of the GE-rich protein family, contain a collagen-binding domain located at the C-terminus^20^. Sequence homology analysis of the C-terminus reveals that four cysteine residues are completely conserved, although the amino acid identity in this region is only around 89% between each protein^20^. As blood feeding behavior such as probing time length and salivary gland antihemostatic activities vary among *Anopheles*, *Aedes* and *Culex* mosquitoes^25^, it would be interesting to examine the relationship between the collagen-binding activities of the GE-rich protein family members and the blood feeding behaviors among the various mosquito species. Because the GE-rich domain in AAPP possesses the characteristic feature of Ca^2+^-binding activity, thus it can prevent a collagen-induced increase in intracellular Ca^2+^, which is the second messenger in the platelet activation cascade^26^, and has evolved to recruit translocating platelets into the developing aggregates. Additionally, AAPP may act as a Ca^2+^ chelator after its bind to collagen, resulting in enhanced inhibition of platelet aggregation.

Reducing the abundance of Aegyptin at the protein level using transgenic RNAi^16^ showed similar results to the ones we have described here. In contrast to silencing the *Aegyptin* gene, our TG mosquitoes produced AAPP without collagen-binding activity, at similar amounts as AAPP in the WT mosquitoes. Although the ELISA showed that the scFv secreted through the saliva causes almost complete abrogation of AAPP-collagen binding activity from each line of TG mosquito (Table 1), further investigation will be required to validate the loss of the function of AAPP caused by 8H7scFv. Despite the shortage of collagen-binding activity, AAPP in TG mosquitoes may still retain its Ca^2+^-binding activity in the GE-rich region. We found no apparent phenotypic difference between AAPP protein inactivation and Aegyptin knock-down, suggesting other functions such as participation in oligomerization which elucidated in immunoblotting. AAPP multimers and bands with high molecular weights were recognized by the anti-AAPP mAb in the salivary gland lysates (Figs 2D and 3A). The oligomerization might enable AAPP to increase the number of collagen binding sites. Clearly, further studies are needed to elucidate the precise function of the Ca^2+^ binding properties of AAPP.

We further examined whether the behavioral changes in the TG lines would affect sporogonic development of the malaria parasites. No significant reductions in oocysts number were observed in the TG mosquitoes following their blood meals on *P. berghei*-infected mice, indicating that oocyst formation is not likely to be affected by the blood meal size. This result suggests that inactivating a single saliva protein does not affect sporogonic development of the parasite. A recent study also reported that the number of oocysts developing in the mosquito midgut depends on the density of malaria gametocytes in the host’s blood^27^. Nevertheless, it is also of great interest to address the effect of saliva protein on malaria transmission from mosquito to vertebrate host. It has been documented that in hematophagous arthropods, the saliva components not only facilitate in the acquisition of the blood meal but also modulate vertebrate immune responses resulting in a local microenvironment that favors the establishment of a vector-borne disease^28^. Therefore, vertebrate host immunity that blocks the pharmacological action of the salivary constituents has the potential to affect a vector’s feeding ability and transmission of vector-borne pathogens. It has been reported that transgenic *An. stephensi* mosquitoes producing extremely low amounts of saliva enabled to ingest blood and the resulting phenotype showed a significant reduction in the ability to transmit malaria parasites and salivary glands homogenate exhibited reduced exflagellation *in vitro*^24^. This raises the possibility of the development of a saliva protein-based vaccine that targets malaria transmission stages^29^.

In conclusion, we confirmed that AAPP, a predominant salivary protein, plays an important role in facilitating blood feeding in *An. stephensi*, but the interruption observed in the blood feeding behavior of this mosquito did not affect its malaria vectorial capacity (sporogonic development) in our laboratory model. Our studies reinforce current knowledge about collagen-induced platelet aggregation inhibition in hematophagous arthropods salivary glands and its crucial role in successful blood meal acquisition. Our transgenesis-based protein inactivation and protein-protein interaction methods provide an exclusive opportunity to clarify the complex interactions occurring between multifunctional saliva proteins and host homeostasis or pathogen transmission *in vivo*.

## Materials and Methods

### Ethics statement

All animal care and handling procedures were approved by the Animal Care and Ethical Review Committee of Kanazawa University (No. 22118–1) and Jichi Medical University (No. 16092), Japan. All experimental procedures were performed in accordance with the Guidelines for Animal Care and the Ethical Review Committee of Kanazawa University (No. 22118–1) and Jichi Medical University (No. 16092), Japan. All efforts were made to minimize animal suffering during the experiments.

### Animals, parasites, and mosquitoes

The female BALB/c and slc: ICR mice obtained from Japan SLC (Hamamatsu, Shizuoka, Japan) were used in all experiments at 7–8 weeks of age. *P. berghei* ANKA parasites constitutively expressing green fluorescent protein (GFP)-*P. berghei* (Pb-conGFP)^30^ were maintained by cyclical passaging through slc: ICR mice and *An. stephensi* (SDA 500 strain) according to a standard protocol^14,31^.

### AAPP-collagen binding inhibition assays

The AAPP-collagen binding inhibition assay with 8H7 and 28B8 mAbs was performed by ELISA and related methods used are described in the Supplementary Materials and Methods S1.

### Protein separation and Ca^2+^ binding assays

Recombinant proteins were solubilized with Laemmli buffer containing 2% 2-mercaptoethanol (2-ME) and then boiled for 5 min. The proteins were separated by 10% sodium dodecyl sulfate polyacrylamide (SDS-PAGE), and the gels were either stained using the Rapid CBB KANTO Kit (KANTO Chemical Co., Ltd, Tokyo, Japan) or the Stains-All metachromatic cationic carbocyanine dye (WAKO Chemical Inc., Tokyo, Japan), as described in the literature^32^. A Ca^2+^ binding assay was performed as described in the literature^33^. Briefly, a nitrocellulose membrane was pre-equilibrated for 30 min in a buffer solution consisting of 60 mM KCl, 5 mM MgCl_2_ and 10 mM imidazole-HCl (pH 6.8) and then air-dried at room temperature for 2 h. The recombinant proteins, ovalbumin, and bovine brain calmodulin, each at a concentration of 1 μg/µl per recombinant protein (Calbiochem, San Deigo, CA, USA) were dissolved in ddH_2_O and then spotted in triplet onto the membane in equimolar amounts. Calmodulin was used as the reference Ca^2+^-binding protein, and a 1:15 dilution of it was also spotted onto the membrane as described above. The membrane was air-dried at room temperature for 2 h and then incubated with 10 ml of a buffer containing 1 μCi/ml ^45^Ca^2+^ (Amersham Biosciences Ltd, Buckinghamshire, UK) for 1 h. After incubation, the membrane was rinsed for 5 min with the buffer and air dried at room temperature for 2 h before autoradiography and exposed to X-ray film for 9 days at −70 °C. Densitometric analysis of the autoradiograph was performed using the SCAN ANALYSIS software program (BIOSOFT, Great Shelford, Cambridge, UK).

### *Minos* vector construction and embryo microinjections

The gene fragments encoding 8H7 V_H_ and V_L_ have already been cloned from 8H7 hybridoma cells^23^. The DNA sequences for the V_H_ and V_L_ genes have been deposited in GenBank^®^ database under the accession numbers AB903029 and AB903030, respectively. The gene encoding 8H7scFv, which consists of the V_H_ and V_L_ genes linked to a hinge sequence encoding (Gly_4_Ser)_3_, was inserted into the EcoRI/SphI restriction sites of pENTR-aappP-mDsRed-SP15-antryp1T^12^ to generate pENTR-aappP-mDsRed-8H7scFv-antryp1T. The pMinos-EGFP-aappP-mDsRed-8H7scFv-antryp1T transformation plasmid was generated by incubating pMinos-EGFP-RfA-F^9^ and pENTR-aappP-mDsRed-8H7scFv-antryp1T in the presence of LR Clonase (Invitrogen, Thermo Fisher Scientific, Waltham, MA, USA), as described previously^9^. The procedures used to microinject the embryos, screen for G_1_ larvae that express EGFP and generate homozygous lines were conducted as described previously^34^.

### Estimation of transgene copy numbers in transformed mosquito lines by qPCR

The copy numbers of the transgenes to be inserted in the three TG mosquito-lines genomes were determined by qPCR using the AAPP promoter (*pAAPP*) and 8H7 gene as the targets for PCR amplification. qPCR related methods used are described in the Supplementary Materials and Methods S2.

### Production of the anti-mDsRed polyclonal antibody

The gene encoding monomeric DsRed (mDsRed) was excised from plasmid pDsRed-monomer-C1 by digestion with NcoI and SalI, and then cloned into the NcoI/XhoI sites of pET32b^35^ to generate pET32b-mDsRed. The recombinant mDsRed protein, created as a fusion protein with Trx, was expressed in *E. coli* and then purified using a Ni-NTA affinity column (Qiagen, Valencia, CA, USA). BALB/c mice were intraperitoneally immunized with the mDsRed protein with Imject^®^ Alum (Thermo Scientific, Waltham, MA, USA) three times at 3-weekly intervals. Three weeks after the last immunization, whole blood from the immunized mice was collected by cardiac puncture and sera was harvested, stored at −20 °C and used later for the immunoblotting experiments.

### Immunoblotting and Ni-NTA pull-down assays

Groups of 20 pairs of female mosquito salivary glands from WT and TG mosquitoes were homogenized using a plastic homogenizer with 50 µl of Laemmli buffer containing 2% 2-ME, and then boiled at 95 °C for 5 min. Each sample was separated on a 12% SDS-PAGE gel and transferred to an Immobilon FL^®^ PVDF membrane (Merck Millipore, MA, USA). Anti-AAPP mAb and anti-mDsRed polyclonal antibody were used as the primary antibodies. Prior to immunoblotting, the membranes were blocked with 5% skimmed milk (WAKO Chemical Inc., Tokyo, Japan) in 0.1% Tween-20 in PBS (PBST). The membranes were incubated with the primary antibodies, washed, probed with a secondary antibody conjugated to IRDye 800 (Rockland Immunochemicals, Gilbertsville, PA, USA), and then visualized under an infrared imager (Odyssey *LI-COR*, Lincoln, NE, USA). For the Ni-NTA pull-downs, homogenates of the dissected salivary glands from both TG and WT mosquitoes were lysed with Cellytic^TM^ M (Sigma-Aldrich, St. Louis, MO, USA). Ni-NTA beads (Qiagen, Valencia, CA, USA) (100 μl aliquots) were added to the mixtures, followed by incubation on ice for 1 h with shaking. The Ni-NTA beads allowed the recombinant proteins to be pelleted, and after washing each pellet with PBS three times with intermittent centrifugation, the proteins were eluted by boiling the preparations in 50 µl of Laemmli buffer with 2% 2-ME, followed by storage at −20 °C until they were used for the immunoblotting experiments.

### Saliva collection

Twenty female mosquitoes (10 days old, never blood feed), both TG and WT, were used for collecting saliva from their proboscis. The salivation protocol and related methods used are described in the Supplementary Materials and Methods S3.

### Quantification of AAPP levels

AAPP in saliva or salivary gland homogenates was quantified by ELISA as described previously^20,23^. Briefly, soluble type-I collagen (0.3 mg/ml diluted in HCl, pH 3.0; Becton Dickinson, Franklin Lakes, NJ, USA) was immobilized in 96-well EIA/RIA polystyrene plates (Corning Inc.; Corning, NY, USA) at 100 µl/well (7.5 µg/ml), blocked with blocking buffer (1% BSA in PBS) for 1 h at room temperature, after which recombinant Trx-AAPP_ex1–4_, saliva solutions or salivary gland homogenates were applied to the collagen-coated plates and incubated at room temperature for 1 h. After washing with excessive PBST followed by PBS (three times each), the anti-AAPP antibody, diluted 2,000 fold in blocking buffer, was added and incubated for 1 h. AAPP–collagen binding was detected using an HRP-conjugated anti-His antibody (Bio-Rad Inc., Hercules, CA, USA).

### Probing time analysis

Probing time in the TG and WT mosquitoes was measured according to the method described previously^24^. Probing time is defined as the time from initial insertion of the proboscis into the host’s skin until the initial observable ingurgitation of blood. Mosquitoes, 5–7 days old, never blood fed, were sugar-starved the night before the tests, and individually caged in polystyrene vials (Thermo Fisher Scientific, Waltham, MA, USA) (70 × 120 mm) with the hole (20 mm diameter) sealed by a cotton net. Four groups of 35 mosquitoes each (TG-03, TG-30, TG-37, and WT) were used. Mice were anesthetized with ketamine (100 mg/kg, intramuscular, i.m.; Daiichi Sankyo, Tokyo, Japan) and xylazine (10 mg/kg; i.m.; Bayer, Tokyo, Japan)^36^. Their backs were shaved and placed in direct contact with the mosquito-containing cages. Probing time were measured for three different naïve mice, alternating the mosquitoes within each group. Mosquitoes that did not initiate probing or showed no interest in the host after 5-min exposure were eliminated from the analysis. Probing-time observations were terminated after 420 s, and the times recorded were used in the analyses. Data were analyzed using Dunnett’s multiple comparisons test vs the control with a 95% confidence interval (CI).

### Prediuresis time analysis

Prediuresis time in TG and WT mosquitoes were determined according to the method described previously^24^. Prediuresis time was defined as the time taken from initial insertion of proboscis into the host skin until the first droplet of prediuresis liquid was observed. Briefly, female mosquitoes (5–7 days old, never blood fed) were individually caged in transparent polystyrene vials and starved overnight before the experiments commenced. The shaved back of an anesthetized naïve mouse made direct contact with a cage containing the mosquitoes. Prediuresis time were measured on three different mice, alternating single mosquitoes from each group. The times recorded were used in the analyses. Data were analyzed using the Dunnett’s multiple comparisons test vs the control with a 95% CI.

### Feeding success for direct feeding and artificial membrane feeding

Feeding success in the TG and WT mosquitoes was determined using anesthetized mice or MFA, as described previously^16^. Four groups (TG-03, TG-30, TG-37, and WT) of 30 5–7-days-old female mosquitoes were caged and deprived of sugar the day before the experiments were performed. Each cage was offered an anesthetized naïve mouse and the mosquitoes were allowed to feed for 10 min. The number of fed and unfed mosquitoes was scored. Females with either their abdomens fully or partially engorged were considered as fed. For the MFAs, the same four groups of mosquitoes (30 females each), were allowed to feed for 10 min on an artificial membrane feeder (Chemglass Life Sciences, Vineland, NJ, USA) covered with a stretched parafilm membrane (Fuji film Corp., Tokyo, Japan). The blood meal consisted of human defibrinated blood. Membrane feeders were kept at 37 °C during feeding, and the mosquitoes were assessed as fed or unfed as per the direct feeding experiments in mice.

### Analysis of the amounts of blood ingested

The blood meal size ingested by the TG and WT mosquitoes from mice or the MFA was assessed. The abdomens of the engorged mosquitoes were dissected and homogenized in 50 μl of PBS (pH 7.4). The homogenates were centrifuged at 10000 × g for 5 min at 4 °C, and the supernatants were collected and kept on ice. Total hemoglobin in their abdomens was quantified using the hemoglobin colorimetric assay kit (Cayman, Ann Arbor, MI, USA). From each sample, 20 μl was added in duplicate to a 96-well microtiter plate followed by 180 μl of kit’s hemoglobin detector. The microtiter plate was incubated while protected from light at room temperature for 15 min, and the absorbance was measured at 560 nm in a microtiter plate reader (Multiskan FC, Thermo Scientific, Waltham, MA, USA).

### Malaria infection assays

TG and WT mosquitoes, 5–7 days after eclosion, were allowed to feed on the same *P. berghei* infected mouse for 30 min. Unfed mosquitoes were counted and discarded. Only fed mosquitoes were kept on a 5% fructose diet at 21 °C. On days 10–12 the midguts were dissected, and the number of oocysts per midgut was counted under a phase contrast microscope. On days 19–21 the salivary glands were dissected, and the number of sporozoites per salivary gland was counted.

### Statistical analysis

Experimental data were analyzed with Prism version 7.0a (GraphPad Software Inc., La Jolla, CA, USA) and plotted as bar graphs or scatter plots. Dunnett’s multiple comparison tests (95% CI) and two-way analysis of variance were performed in the comparisons analysis. *p* values of < 0.05 were considered statistically significant. A *Chi*-square test (95% CI) was used to compare the feeding success calculation. The Kruskal-Wallis test (95% CI) was used to analyze the infection rate.

## Supplementary information

Supplimental Information
