# Supplementary material for: Anopheline antiplatelet protein from mosquito saliva regulates blood feeding behavior
Source: Sci Rep. 2019 Feb 28;9:3129. doi: 10.1038/s41598-019-39960-2 (PMC6395645; doi:10.1038/s41598-019-39960-2)
Supplement: Supplementary file 1 — Supplimental Information [file 41598_2019_39960_MOESM1_ESM.pdf]

Anopheline antiplatelet protein from mosquito saliva regulates blood feeding behavior

Ashekul Islam[1], Talha Bin Emran[1], Daisuke S. Yamamoto[2], Mitsuhiro Iyori[1], Fitri Amelia[1], Yenni Yusuf[1], Ririka Yamaguchi[1], Md. Shah Alam[3], Henrique Silveira[4] & Shigeto Yoshida[1]*

Supplemental Information

AAPP-collagen binding inhibition assays

8H7 and 28B8 anti-AAPP mAbs have been described previously[1]. The collagen-AAPP binding inhibition assay with 8H7 and 28B8 mAbs was performed as described previously[2]. In brief, 96-well enzyme immunoassay (EIA) plates were collagen-coated by immobilization of soluble type-I collagen (0.3 mg/ml diluted with HCl, pH 3.0; Becton Dickinson, Franklin Lakes, NJ, USA) followed by blocking with phosphate-buffered saline (PBS) containing 1% BSA. Serial dilutions of 8H7 and 28B8 mAbs were incubated with 4 nM of thioredoxin (Trx)-$AAPP_{ex3\text{-}4}$[3] for 1 h, and then applied to the collagen-coated plates and incubated for 1 h. Binding of AAPP to collagen was detected using an anti-His Ab conjugated to horseradish peroxidase (HRP) (Qiagen, Hilden, Germany). The AAPP-collagen binding inhibition rate was determined by comparison with the non-mAb control.

Estimation of transgene copy numbers in transformed mosquito lines by qPCR

The copy numbers of the transgenes to be inserted in the three TG mosquito-lines genomes were determined by qPCR using the AAPP promoter (*pAAPP*) and 8H7 gene as the targets for PCR amplification. Genomic DNA (gDNA) from WT mosquitoes was used as the control. One copy of *pAAPP* per genome is expected in the WT mosquitoes and two or more in the TG mosquitoes. gDNA was extracted from whole mosquito larvae using the QIAamp® DNA Blood Mini Kit (Qiagen GmbH, Hilden, Germany) following the manufacturer's instructions. The extracted DNA was stored at −20°C until use. gDNA concentrations were estimated by measuring the nucleic acid absorbance at 260 nm. Sample purity was estimated from the 260/280 nm ratio for each preparation, the concentrations of

which were adjusted to be equal. The pENTR-pAAPP-mDsRed-G4S1-8H7scFv-polyA plasmid DNA harboring the *pAAPP* and 8H7 genes was used to produce a 10-fold diluted standard curve ranging from 1 to 10^6 copies. Quantitative analysis of the gDNA was performed using Real-time PCR with SYBR® Green Premix Ex Taq™ (Takara, Tokyo, Japan) and the following primer sets: pAnSG-F14 (5′-ACGGTTCCGAGGTCCGACGATCGTC-3′) and pAnSG-R3 (5′-AAGCTTCATCGTTTATTCACCTG-3′), p8H7-F1 (5′-CCGAAGCGTCTGATCTACCT-3′) and p8H7-R1 (5′-GGCTGATCTTCAGGGTGAAA-3′). Samples were run in triplicate. Three qPCRs were performed, each with a freshly prepared DNA dilution. Standard curves were generated and the number of copies of each gene in each sample was determined and the average values were calculated.

Saliva collection

Twenty female mosquitoes (10 days old, never blood feed), both TG and WT, were used for the previously described salivation protocol[4] with some modifications. Briefly, the mosquitoes were chilled and immobilized, and each mosquito proboscis was placed into 50 µl PBS (pH 7.4) for 3 min. Saliva in the PBS was observed with a fluorescence microscope (BZ-X700; Keyence Corp., Osaka, Japan) under a DsRed filter and then stored at −20°C until further analysis.

Egg hatchability and adult survivorship

Egg hatchability and the adult survivorship were assessed in the TG mosquitoes and their WT siblings as described in literature[5,6]. Briefly, group of 35 newly eclosed TG males and females were selected and used to set up pairwise mating for each of the 4 experimental lines (TG-03, TG-30, TG-37, and WT). Female mosquitoes were then exposed to anesthetized mouse to obtain a blood meal without time limit. Fed females (n=20) were put individually into oviposition cup and maintained on a 5% fructose meal. Egg deposition was monitored at 72 hours post blood meal and the number of eggs laid by each female was counted and recorded as an index of fecundity, excluding females that laid no eggs. To assess the hatchability, the laid eggs were collected on a wet filter paper and kept in a wet Petri dish. After two days at 27°C, the number of larvae and no hatched eggs was counted with the aid of microscope. The

numbers of larvae hatching from the laid eggs were recorded as a measure of fertility. To estimate adult survivorship, 20 adults from each lines of both sexes were caged separately and fed on 5% fructose or anesthetized mice without time restriction. The total surviving adults in the case was recorded daily till all died. Insectary procedures were standardized to ensure that all mosquitoes were treated similarly.

1. Sugiyama, K. *et al.* The crystal structure of the active domain of *Anopheles* anti-platelet protein, a powerful anti-coagulant, in complex with an antibody. *J Biol Chem* **289**, 16303-16312, doi:10.1074/jbc.M114.564526 (2014).
2. Yoshida, S. *et al.* Inhibition of collagen-induced platelet aggregation by anopheline antiplatelet protein, a saliva protein from a malaria vector mosquito. *Blood* **111**, 2007-2014, doi:10.1182/blood-2007-06-097824 (2008).
3. Hayashi, H. *et al.* Identification of the active region responsible for the anti-thrombotic activity of anopheline anti-platelet protein from a malaria vector mosquito. *Platelets* **24**, 324-332, doi:10.3109/09537104.2012.698430 (2013).
4. Dubrulle, M. *et al.* *Chikungunya* virus and *Aedes* mosquitoes: saliva is infectious as soon as two days after oral infection. *PLoS One* **4**, e5895, doi:10.1371/journal.pone.0005895 (2009).
5. Amenya, D. A. *et al.* Comparative fitness assessment of *Anopheles stephensi* transgenic lines receptive to site-specific integration. *Insect Mol Biol* **19**, 263-269, doi:10.1111/j.1365-2583.2009.00986.x (2010).
6. Li, C., Marrelli, M. T., Yan, G. & Jacobs-Lorena, M. Fitness of transgenic *Anopheles stephensi* mosquitoes expressing the SM1 peptide under the control of a vitellogenin promoter. *J Hered* **99**, 275-282, doi:10.1093/jhered/esn004 (2008).

Supplementary Figures

Figure S1

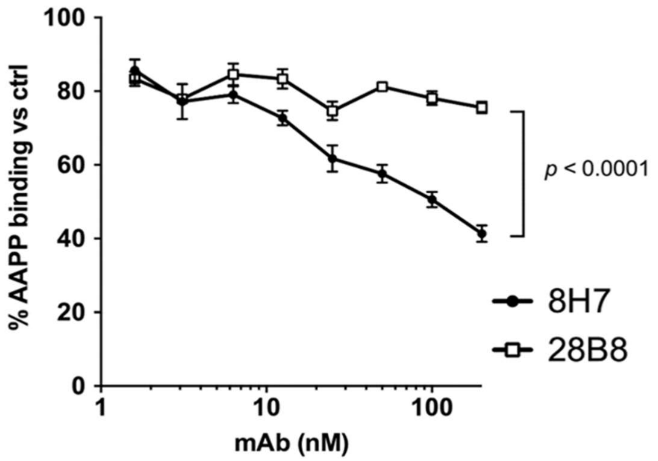

Figure S1. Specific inhibition of collagen binding of AAPP by 8H7 mAb. Serially diluted 8H7 mAb or 28B8 mAb was pre-incubated with Trx-AAPP$_{ex3-4}$ and then applied into the collagen-coated plates. Bound AAPP was detected by anti-His antibody conjugated with HRP. Data are shown as mean ± SD of a percentage of AAPP-binding units (OD$_{414nm}$) compared with non-mAb control wells (ctrl). Two-way ANOVA with Sidak's multiple comparisons test (n = 4).

Figure S2

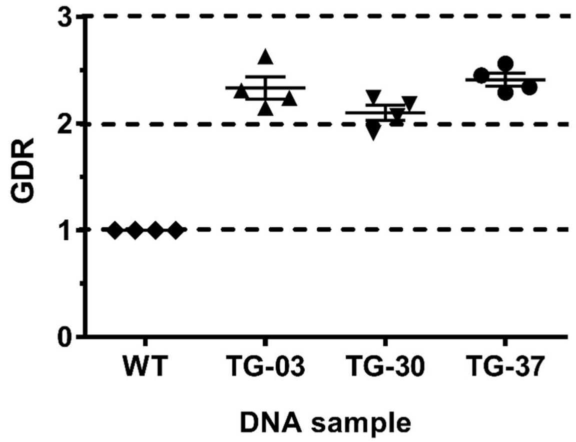

Figure S2. Gene Dose Ratio (GDR) of the *pAAPP* gene in WT and TG mosquito genome. DNA samples were obtained from pooled larvae samples from wild type (WT) and transgenic (TG) mosquito lines. GDR values are relative to the number of *pAAPP* gene copies in each sample. Error bars represent the SE from three replicates.

Figure S3

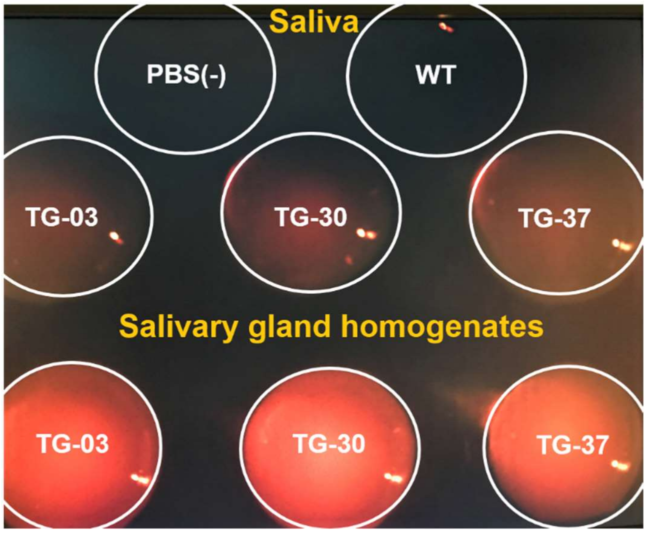

Figure S3. Observation of saliva under fluorescence microscope. Live female mosquitoes from both WT and TG mosquitoes (10 days old, never blood feed, n=20) were chilled. Each mosquito was held by forceps and embedded its proboscis into PBS (pH-7.4) for 3 min to collect saliva. The saliva solutions or dissected salivary glands homogenate were observed under fluorescence microscope BZ-X700 (Keyence Corp. Osaka, Japan) in a DsRED filter set.

Figure S4

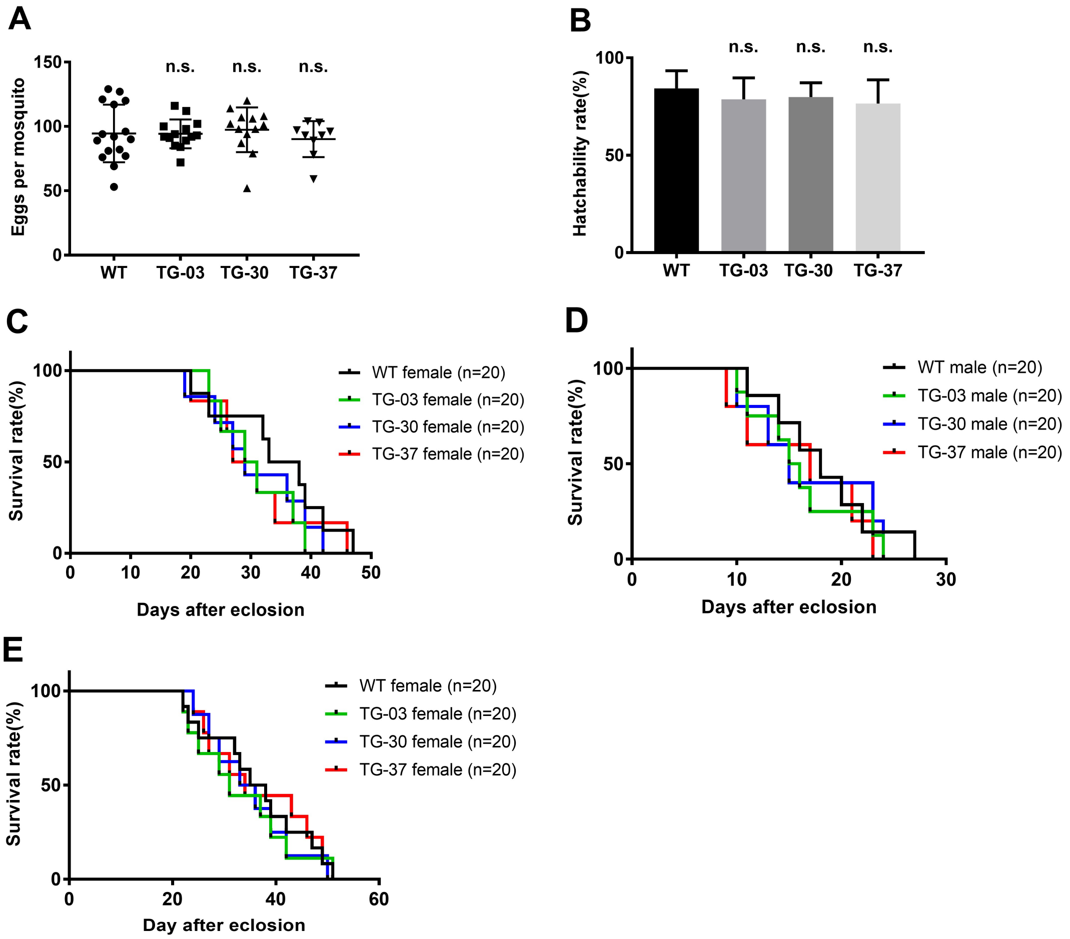

Figure S4. Comparison of egg hatchability and longevity between TG and WT mosquitoes. (A, B) Fecundity (A) and hatchability (B) were investigated in both TG and WT mosquitoes that fed on mice without time restriction. Statistical analyses of fecundity and hatchability were performed using the Dunnett's multiple comparisons test with 95% CI. No significant difference was observed in females between TG and WT mosquitoes. Horizontal bars represent the median value. n.s., not significant. (C, D, E) Longevity of female (C) and male (D) mosquitoes were investigated under the sugar feeding condition or female mosquitoes (E) fed on mice without time restriction (n=20). Statistical analyses of survival curves were performed using the log rank test with 95% CI. No significant difference was observed in male or female mosquitoes between TG and WT lines.
